# Predicting Antigenic Distance from Genetic Data for PRRSV-Type 1: Applications of Machine Learning

**DOI:** 10.1128/spectrum.04085-22

**Published:** 2022-12-13

**Authors:** Dennis N. Makau, Cinta Prieto, Francisco J. Martínez-Lobo, I. A. D. Paploski, Kimberly VanderWaal

**Affiliations:** a Department of Veterinary Population Medicine, College of Veterinary Medicine, University of Minnesota, Minneapolis, USA; b Departamento de Sanidad Animal, Facultad de Veterinaria, Universidad Complutense de Madrid, Madrid, Spain; c Departament de Ciència Animal, ETSEA-Universitat de Lleida, Lleida, Spain; Johns Hopkins Hospital

**Keywords:** cross-protection, seroneutralization, immunodominant sites, immunogenicity, immune response, machine learning, bioinformatics

## Abstract

The control of porcine reproductive and respiratory syndrome (PRRS) remains a significant challenge due to the genetic and antigenic variability of the causative virus (PRRSV). Predominantly, PRRSV management includes using vaccines and live virus inoculations to confer immunity against PRRSV on farms. While understanding cross-protection among strains is crucial for the continued success of these interventions, understanding how genetic diversity translates to antigenic diversity remains elusive. We developed machine learning algorithms to estimate antigenic distance *in silico*, based on genetic sequence data, and identify differences in specific amino acid sites associated with antigenic differences between viruses. First, we obtained antigenic distance estimates derived from serum neutralization assays cross-reacting PRRSV monospecific antisera with virus isolates from 27 PRRSV1 viruses circulating in Europe. Antigenic distances were weakly to moderately associated with ectodomain amino acid distance for open reading frames (ORFs) 2 to 4 (ρ < 0.2) and ORF5 (ρ = 0.3), respectively. Dividing the antigenic distance values at the median, we then categorized the sera-virus pairs into two levels: low and high antigenic distance (dissimilarity). In the machine learning models, we used amino acid distances in the ectodomains of ORFs 2 to 5 and site-wise amino acid differences between the viruses as potential predictors of antigenic dissimilarity. Using mixed-effect gradient boosting models, we estimated the antigenic distance (high versus low) between serum-virus pairs with an accuracy of 81% (95% confidence interval, 76 to 85%); sensitivity and specificity were 86% and 75%, respectively. We demonstrate that using sequence data we can estimate antigenic distance and potential cross-protection between PRRSV1 strains.

**IMPORTANCE** Understanding cross-protection between cocirculating PRRSV1 strains is crucial to reducing losses associated with PRRS outbreaks on farms. While experimental studies to determine cross-protection are instrumental, these *in vivo* studies are not always practical or timely for the many cocirculating and emerging PRRSV strains. In this study, we demonstrate the ability to rapidly estimate potential immunologic cross-reaction between different PRRSV1 strains *in silico* using sequence data routinely collected by production systems. These models can provide fast turn-around information crucial for improving PRRS management decisions such as selecting vaccines/live virus inoculation to be used on farms and assessing the risk of outbreaks by emerging strains on farms previously exposed to certain PRRSV strains and vaccine development among others.

## INTRODUCTION

Porcine reproductive and respiratory syndrome (PRRS) continues to be one of the leading causes of economic losses and reduced pork production in most major pork-producing countries (1, 2). PRRS, caused by a positive sense single-stranded RNA virus (PRRSV) (3–6), causes abortion and stillbirths, pneumonia, preweaning mortalities, and stunted growth in sows, piglets, and growing pigs, respectively (1–4, 7, 8). Originally classified as two broad genotypes, PRRSV type 1 and type 2 that referred to the so-called North American and European strains, PRRSV is now classified as Betaarterivirus suid 1 (PRRSV1) and Betaarterivirus suid *2* (PRRSV2) in the order *Nidovirales* (9–11). Evolutionary diversification in PRRSV1 resulted in further classification of the species into four main subtypes (subtypes 1 to 4) circulating in Europe (10, 12).

The genetic and antigenic diversity in PRRSV has greatly contributed to challenges in controlling PRRS outbreaks on farms using vaccines or live virus inoculation. Relatively suboptimal/poor cross-protection between PRRSV isolates, especially against heterologous strains, is a common issue in immunization (13, 14), yet there are no robust criteria for defining strains as heterologous from an antigenic perspective. Thus, the classification of PRRSV strains as homologous versus heterologous predominantly relies on the characterization of viral genomes or portions of the genome to distinguish between variants (i.e., restriction fragment length polymorphisms, lineages/subtypes, or genetic distance) (15–18). However, inadequate knowledge of the impact of genetic differences in viral populations makes it difficult to infer host immune responses after exposure to genetically distinct viruses (19). In addition, genetic differences do not always translate to antigenic change, and although our knowledge of the functionality of the PRRSV genome is incomplete, some genomic regions and specific amino acid positions are clearly more important than others from an antigenic standpoint (20–22).

Although numerous efforts have been made to correlate specific genetic differences to the clinical presentation of disease (8, 12, 23–26) and host immune response (27, 28), the question of cross-protection remains largely unanswered. Stronger anamnestic immune responses, reducing clinical symptoms or sometimes preventing reinfection, have been documented when pigs were challenged with homologous viruses, and less consistent immune responses were observed when challenged with heterologous viruses (27, 29, 30). The quality of the immune response is associated with the ability of the virus to mobilize the B-cell response and stimulate the production of neutralizing antibodies (a function of neutralizing epitopes on the viral surface) (20, 31). As such, changes/mutations in the genome that affect linear or conformational epitopes may influence immune response and ability to confer cross-protection to homologous or heterologous viruses (32). Structurally, the PRRSV genome is divided into structural and nonstructural portions comprised of 10 open reading frames (ORFs) (33). Although neutralizing epitopes and antigenic sites have not been exhaustively described for different segments of the PRRSV genome, some portions have been identified as critical players in the production of antibodies. Using different approaches, including monoclonal antibody analysis (34, 35), analysis of chimeric mutants, peptide analysis (36–38), and mutagenesis (39), glycoprotein 2 (GP2), GP3, GP4, GP5, and M proteins have been associated with induction of neutralizing antibodies.

At the population level, substitutions in antigenic sites have been hypothesized to play a role in the cyclic emergence and spread of PRRSV variants with differences in known antigenic sites (15). The cyclic emergence and spread of new lineages pose a significant challenge to disease management, with widespread dissemination of emerging variants attributable to animal movements (26, 40, 41). In *in vitro* studies, cross-neutralization assays (serum neutralization [SN] assays) have been used to quantify the level of heterologous and homologous cross-reactivity between different viruses and antibodies generated against them (42). Genetic differences between viral genomes, especially in antigenic sites and epitopes, can be analyzed to correlate genetic changes with variations in host-virus interactions and immune response.

Hemagglutinin-inhibition (HI) assays have been used extensively in influenza studies to estimate antigenic differences between strains based on the hemagglutinin (HA) gene of the virus, which codes for an immunogenic glycoprotein (43). Given the role of HA in pathogenesis and immune response, differences in levels of cross-reaction between strains have been used to estimate how well host immunity against one strain could protect against infection by a second strain and have also been used in the evaluation of vaccine-mediated immunity (44). As such, genetic differences within the HA gene are generally considered responsible for the differences in antigenic phenotypes between viral strains (simply described as antigenic distances between viruses) (45). Outputs from such HI/cross-reactivity assays have been used to develop *in silico* predictive models combining sequence data and machine learning algorithms to predict antigenic differences among viruses (46, 47). Genomic differences between PRRSV and influenzas notwithstanding, similar cross-reactivity data from SN assays can be combined with machine learning algorithms and sequence data for already characterized genes of PRRSV to estimate the antigenic distance between viruses. Further, point differences in these genes can be examined in correlation with differences in antigenic phenotypes to infer how those mutations may influence host immune responses and, potentially, cross-protection *in vivo*. Similar approaches have been applied to other human viruses (47, 48).

Therefore, the objectives of this study were to develop an algorithm to estimate the likelihood of serum-virus cross-protection between PRRSV-1 viruses and identify important amino acid sites that influence antigenic variability between viruses. Further, we investigated how specific differences in those amino acid sites contributed to antigenic variability between the viral isolates. The ability to distinguish between antigenic traits of PRRSV cocirculating in the swine population and estimate potential cross-protection between viruses *in silico* will support decision-making by producers, practitioners, and vaccine developers on the selection and application of vaccines or live virus inoculation for PRRS management.

## RESULTS

### Descriptive analysis.

**FIG 1:**
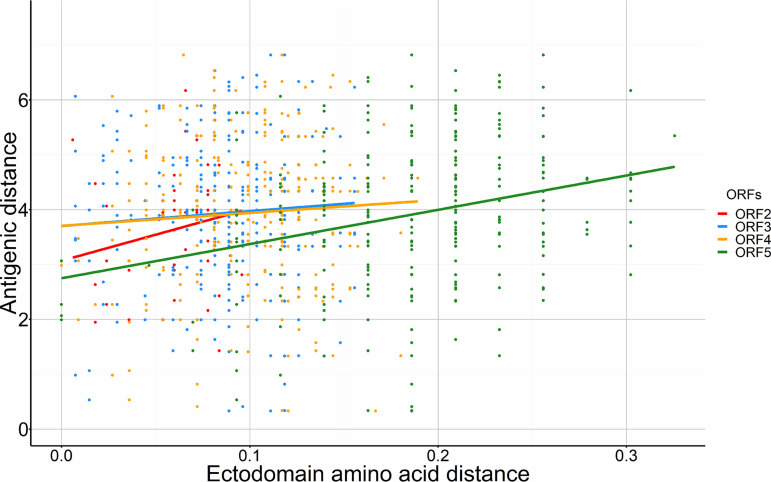
Scatter and line plot depicting correlation between the ORF-specific ectodomain amino acid p-distances and antigenic distances.

Pairwise adjusted antigenic distance ranged from −2.7 to 7.5 units, with a mean (±SD) 3.8 ± 1.5, respectively. Compared to ORFs 2 to 4, the mean pairwise amino acid distance was highest in ORF5 (0.19 ± 0.05) and was moderately correlated with antigenic distance between the serum-virus pairs (Spearman’s correlation coefficient = 0.3, *P* < 0.01) (Table 1 and Fig. 1). After excluding 12 observations with negative antigenic distance from further analysis, the mean (±SD) antigenic distance was 3.9 ± 1.5. For the ORFs 3 to 5 data set (*n* = 612), antigenic distance was categorized into high antigenic distance (*n* = 305) and low antigenic distance (*n* = 307), splitting at the median (3.9). When ORF2 sequence data were added, the total number of pairs was 314, with 146 classified as high and 168 low antigenic distance.

**TABLE 1:** Summary statistics of ORF-specific amino acid distance (full and ectodomains) and Pearson’s correlation with antigenic distance^*a*^

ORF section	Pairwise amino acid distances			Correlation with antigenic distance^*a*^	Correlation *P* value
	Range	Median	Mean ± SD		
2-Full	0–0.133	0.080	0.078 ± 0.03	0.35	0.01
2-Ectodomain	0–0.096	0.063	0.058 ± 0.02	0.16	0.248
3-Full	0–0.195	0.116	0.106 ± 0.04	0.04	0.451
3-Ectodomain	0–0.156	0.089	0.085 ± 0.04	0.03	0.557
4-Full	0–0.170	0.098	0.094 ± 0.03	0.01	0.825
4-Ectodomain	0–0.189	0.099	0.096 ± 0.04	0.04	0.496
5-Full	0–0.185	0.098	0.099 ± 0.03	0.08	0.131
5-Ectodomain	0–0.326	0.186	0.190 ± 0.05	0.25	<0.001
All ORFs-Full	0–0.511	0.178	0.175 ± 0.17	0.03	0.435
All ORFs-Ectodomain	0–0.6203	0.266	0.213 ± 0.20	0.083	0.040

a Spearman’s correlation rho(ρ) between raw p-amino acid distance and antigenic distance.

### Gradient boosting model performance and predictions.

Among the ORF-specific models, ORF5 performed with the highest accuracy of 0.75 (95% confidence interval [CI], 0.72 to 0.79) and specificity (0.81); other ORF-specific models were not distinctly different from each other. For models combining different ORFs, the model combining ORFs 2 to 5 performed best (accuracy = 0.80; 95% CI, 0.76 to 0.85; sensitivity = 0.86; specificity = 0.75; Table 2). The positive and negative predictive values for this model were 0.80 and 0.83, respectively, with a balanced accuracy of 0.81.

**TABLE 2:** Summary of performance of ORF specific and combined ORF models for ORFs 2–5^*a*^

No. Observations	No. features	Model	Sensitivity	Specificity	Accuracy	95% Confidence Interval	NPV	PPV
612	44	ORF5	0.70	0.81	0.75	0.72–0.79	0.73	0.78
612	118	ORF4	0.74	0.75	0.75	0.71–0.78	0.75	0.75
612	136	ORF3	0.75	0.73	0.74	0.70–0.77	0.74	0.73
314	148	ORF2	0.72	0.76	0.74	0.69–0.79	0.76	0.72
Combined ORF models								
**314**	**446**	**ORF2–5**	**0.86**	**0.75**	**0.81**	**0.76**–**0.85**	**0.83**	**0.80**
612	298	ORF3**–**5	0.72	0.80	0.76	0.73–0.80	0.74	0.78
314	192	ORF2 and 5	0.86	0.73	0.80	0.75–0.84	0.82	0.79

a The antigenic distance classes for models for ORFs 3, 4, 5, and 3–5 were 305 high and 307 low, while for ORFs 2, 2 and 5, and 2–5, they were 146 high and 168 low. NPV, negative predictive value; PPV, positive predictive value. The best model used in subsequent analysis is bolded for easier reference.

**FIG 2:**
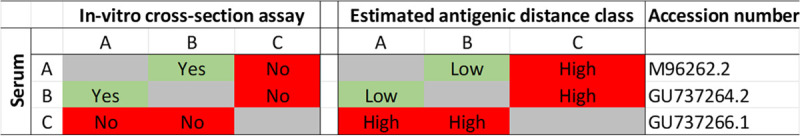
ORF4 model estimation of antigenic classification for two wild isolates isolated in Belgium and one vaccine strain.

To evaluate the performance of our models on external field data, we applied the ORF4-specific model to data generated by Vanhee et al. (49) from PRRSV1 isolates. In that study, they focused on determining the effects of genetic differences in ORF4 on cross-reaction and cross-recognition between sera-virus reactions involving three isolates (virus A, Lelystad vaccine prototype accession no. M96262.2, and two wild strains: virus B, accession no. GU737264.2, and virus C, GU737266.1). The pairwise amino acid distance between all three isolates was 10.7 to 12.9%, thus all would typically be considered genetically heterologous from one another. However, two of the viruses (A and B) cross-reacted with each other, whereas virus C did not cross-react with either of the others. Using the ORF4 model (only ORF4 sequences were available), we correctly classified antigenic distance between the isolates corresponding to the study’s *in vitro* assay findings (Fig. 2).

For each ORF-specific model, specific amino acid sites were ranked according to their importance for model performance to identify influential amino acid sites in each ORF. Briefly, the five highest-ranked amino acid sites were 47, 67, 100, 127, 164 in ORF2; 30, 72, 81, 102, 155 in ORF3; 59, 68, 79, 96, 128 in ORF4; and 36, 49, 101, 105, 106 in ORF5 (Fig. S1a to d in the supplemental material).

**FIG 3:**
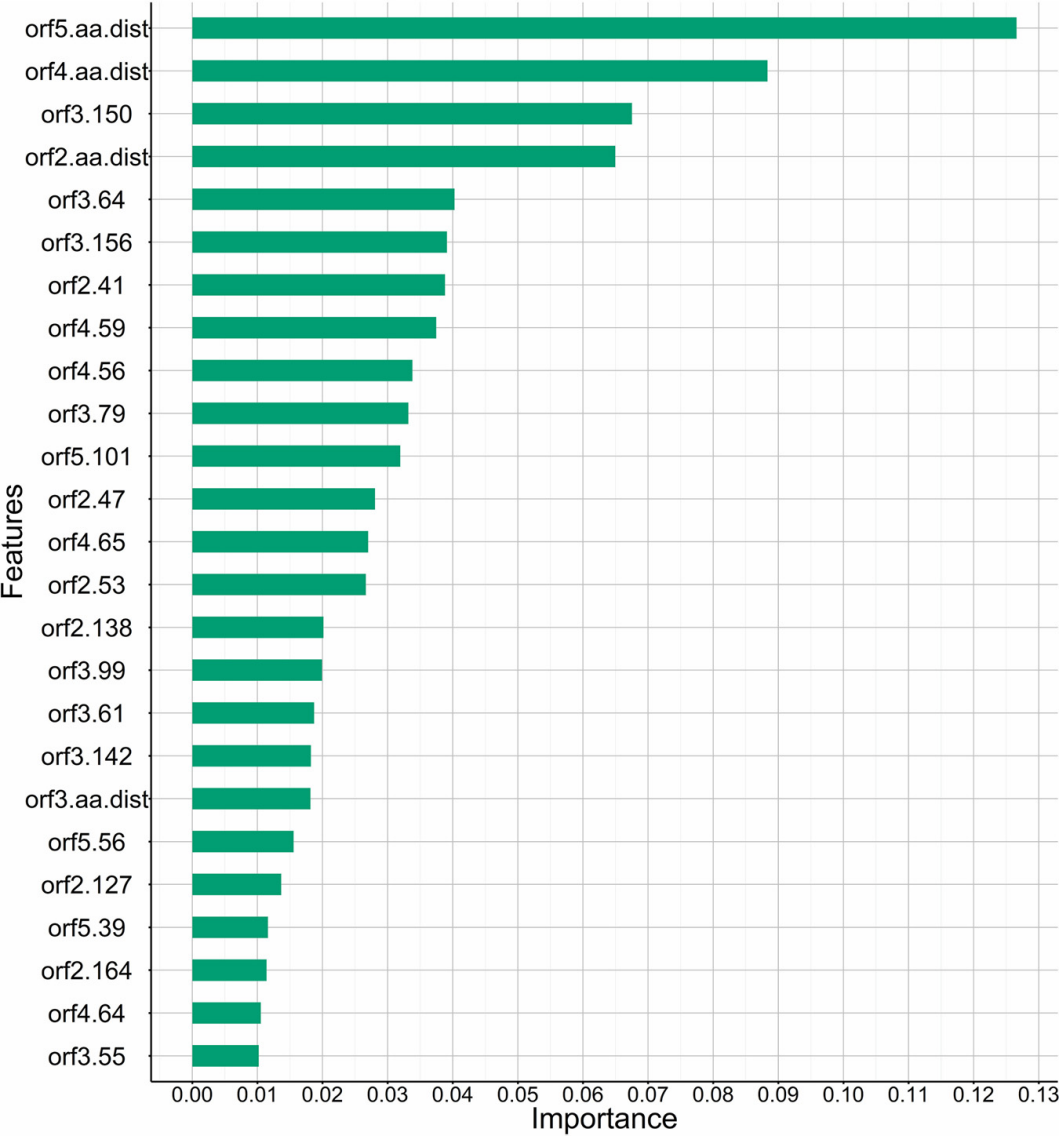
Importance plot for top 25 model predictors (gain ≥0.01) from the best model combining ORFs 2 to 5. Feature labels (*y* axis) are predictors in the models, with the prefix indicating the ORF and the suffix indicating either the amino acid in the ectodomain (aa.dist) or the specific amino acid site. Predictors are ranked based on overall importance considering gain (relative contribution of a variable to model accuracy).

**FIG 4:**
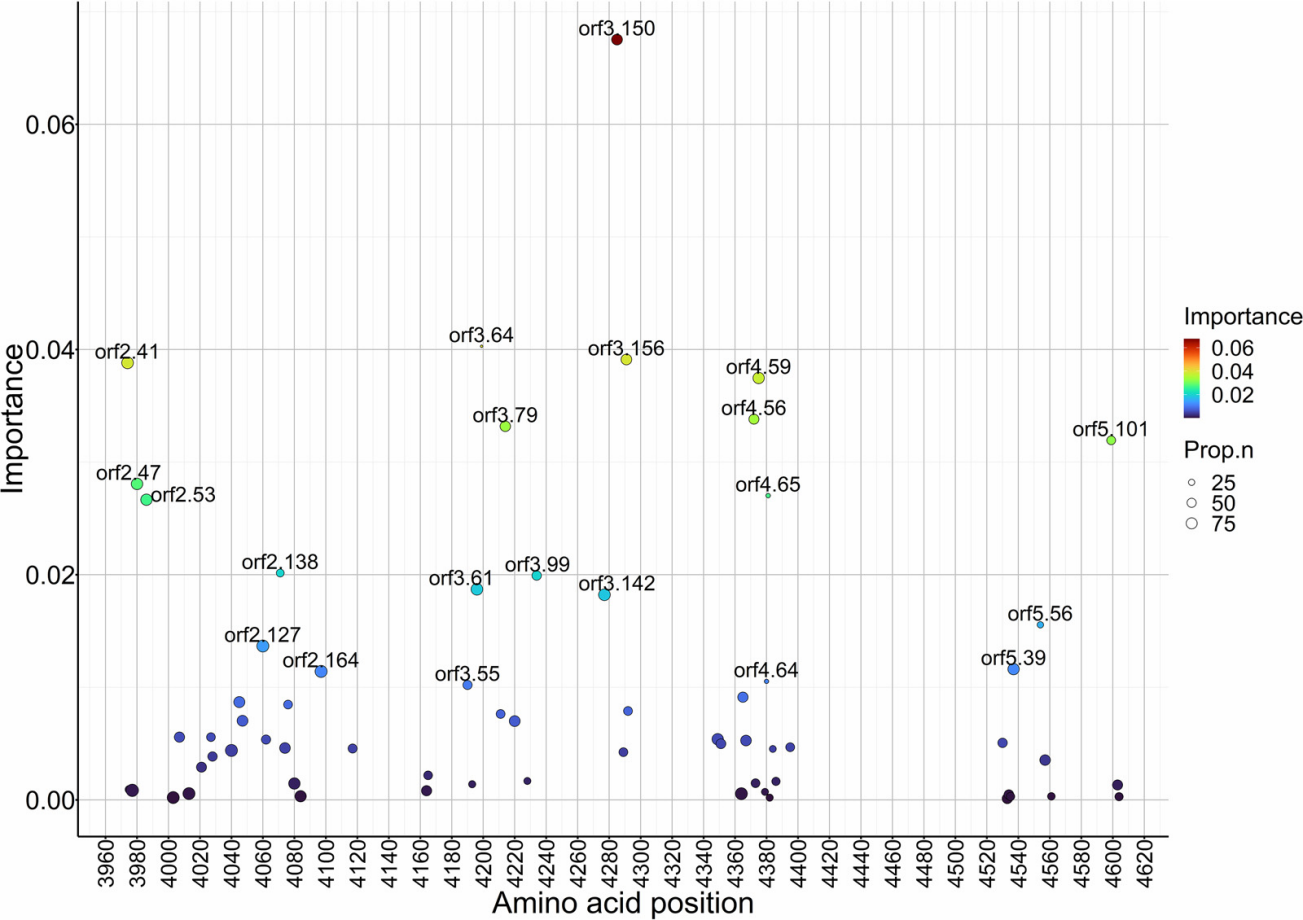
Relative importance (*y* axis) and proportional abundance of polymorphisms (size) in different amino acid sites in ORFs 2 to 5. The *x* axis shows the continuously numbered amino acid position across the PRRSV genome. The importance scale is based on a gradient boosting model gain (relative contribution of a variable to model accuracy).

In addition, using outputs from the best combined ORFs model (ORFs 2 to 5 model), we identified predictors with importance ≥0.01 (*n* = 25), which included 21 amino acid sites as well as ectodomain amino acid distance of each ORF (Fig. 3). Interestingly, ORF5 amino acid distance in the ectodomain was by far the most important predictor, although ORF4 and ORF2 were also highly ranked. The percentage of serum-virus pairs with polymorphisms at the highly ranked specific amino acid sites mostly ranged between 50 and 75% of the data set (*n* = 314) (Fig. 4 and Table S4), suggesting that these sites are all hypervariable. Other polymorphic amino acid sites in the ectodomain regions of ORFs 2 to 5 were estimated to have minimal effect on the model accuracy and ranked low in their importance (Fig. 4; Fig. S1e).

### Multimodal regression quantification effect of site-wise differences in specific amino acids.

**FIG 5:**
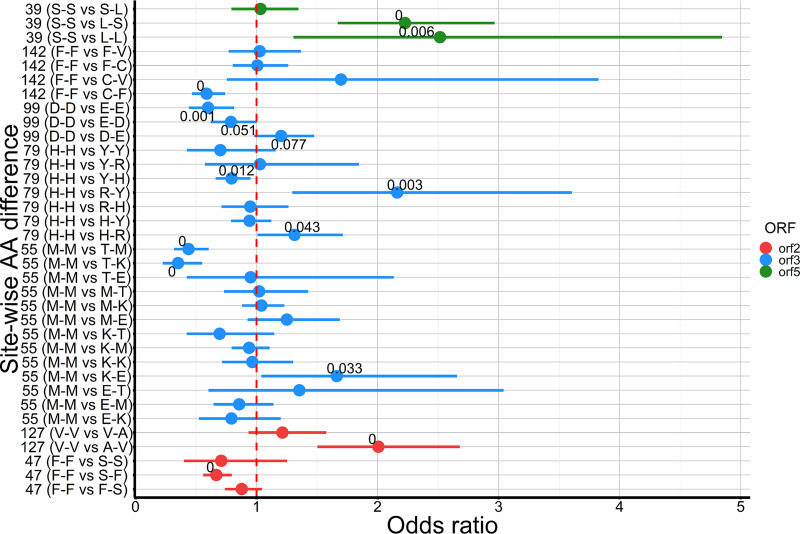
Odds ratios of specific serum-virus site-wise amino acid (AA) differences compared to the no site-wise difference reference (listed first in the *y* axis labels). Error bars represent 95% confidence interval for specific odds ratios, and point labels on the plot indicate the *P* value (only *P* < 0.1 are labeled). Vertical red line demarcates odds ratio of 1 for easier interpretation of significant versus nonsignificant odds ratios.

While the gradient boosting model allowed us to identify sites at which polymorphisms were associated with antigenic distance, it did not allow us to investigate the nature of the specific amino acid differences at those sites. In the multimodal regression, we focused on the specific amino acids that were substituted at each site, allowing us to address the question of how much more likely a sera-virus pair would have a high antigenic distance if they had a specific change at that site (i.e., A→V) versus the reference (i.e., no difference, A→A/V→V). From the multimodal inference analysis, seven amino acid sites were ranked highest from the averaged models (ORF2 = 47, 127; ORF3 = 55, 79, 99, 142; ORF5 = 39). Specific site-wise amino acid differences in ORFs 2, 3, and 5 were significantly associated with classification into high or low antigenic distance (Fig. 5). For example, in ORF5 site 39, when the serum had a leucine and the virus had serine (i.e., the serum-virus pair had a L-S amino acid difference), the odds of high antigenic distance between those pairs were more than twice as high compared to a pair where both the serum and virus had serine in that site (S-S, *P* < 0.05). Similar statistically significant effects were observed on ORF3 site 79 (H-H versus R-Y and H-H versus H-R) and ORF2 site 137 (V-V versus A-V) (*P* < 0.05) (Fig. 5).

## DISCUSSION

In this study, we developed a model to estimate antigenic distance between PRRSV1 isolates using classification-based machine learning algorithms. Using gradient boosting models, we can predict an approximate antigenic dissimilarity between pairs of viruses, which adds to a growing body of research on influenza and other human viruses utilizing similar methods (47, 48, 50–53). By examining amino acid sequences of characterized glycoproteins associated with induction of host immune response, we demonstrate that *in silico* methods can be used to approximate differences in antigenicity for PRRSV. Additionally, we reaffirm that there are some immunodominant sites in the PRRSV ectodomain regions. The ability to estimate differences in antigenicity between viruses *in silico* will be beneficial for vaccine and live virus inoculum selection by swine producers and pharmaceutical companies, as well as aid our ability to understand how immunity plays into the emergence and cocirculation of multiple strains in swine populations (54).

By examining correlations between antigenic distances relative to ORFs 2 to 5 that encode antigenically important glycoproteins (55–57), we observed a positive correlation between ectodomain amino acid distance and antigenic distance, albeit weak for ORFs 2 to 4 and moderate for ORF5 (Fig. 1). Divergent views of this association have been presented by previous studies; e.g., some maintain that genetic variation in ORF5 neither predicts nor explains immunological protection (58, 59), whereas others suggest that genetic variation (genetic distance) in ORFs 2 to 6 influences susceptibility to neutralization (60) and certain mutations in ORF5 support evasion of neutralizing antibodies (61). Our data suggest that genetic distance can inform antigenic distance and cross-reactivity/neutralization between viruses, particularly if the ectodomain is focused on. Host immune responses are primarily triggered by exposure to antigens, some of which are located on the surface of the virus (ectodomains). Some of these exposed ectodomains bear epitopes against which antibodies generated by the host bind to neutralize the virus, hence the higher correlation between antigenic distance and amino acid distances of ectodomains compared to the full ORF distance (summarized in Table 2). In addition, our results statistically demonstrate the association of antigenic distance with particular site-wise amino acid differences, which may be overshadowed when using genetic (or amino acid) distance as the sole measure of genetic difference.

From the gradient boosting analysis, the performance of the combined ORFs 2 to 5 model in predicting antigenic distance between serum and viral isolates was higher than each individual ORF model (Table 2), which provides supporting evidence for the multigenic nature of the interactions between the viral genome and host immune response (60). While certain genes (like ORF5) may be more immunogenic, the role of other ORFs (2 to 4) in immune induction and response cannot be ignored (36, 60). Specific sites identified in our data set are closely located or part of neutralizing epitopes or glycosylation sites of interest in the PRRSV1 genome. For example, one of the immune evasion mechanisms of PRRSV is through glycan shielding (*N*-glycosylation) at certain sites of the genome (21). Substitutions of amino acids in certain sites may result in the loss or gain of *N*-glycosylation sites affecting viral neutralization, infectivity, and host immune response (21, 39, 62, 63). Although the structural characteristics of PRRSV1 have not been thoroughly documented, some studies have proposed that glycosylation sites exist in ORF3 positions 50, 130, and 152 among others and that there is a neutralizing epitope between positions 57 and 73 in ORF3 (20), which are close to some of the important sites identified by our model. In ORF5, the neutralizing epitope in this region is found between positions 38 and 46 (20), which includes site 39 identified by our model.

From the multimodal analysis, we showed that certain amino acid changes were more associated with the odds of a serum-virus pair having high antigenic dissimilarity than others. This effect on antigenic distance could be associated with the physical or chemical properties of the amino acid affecting protein folding or the effect of such differences on *N*-glycosylation. As for other viruses, mutations/substitutions in certain amino acid sites (also referred to as immunodominant sites) may result in changes in epitope structure or complete hiding of an epitope, hence limiting or impairing host immune response and translating to a change in immunogenic phenotype (46, 64–68). For PRRSV, although the ectodomains have been documented to present epitope binding sites, characterization of the role of amino acid substitutions in specific sites in immune response, especially for PRRSV1, has not been exhaustive. Our model found that serine (polar)-leucine (nonpolar) differences in site 39 in ORF5, which is part of the neutralizing epitope, resulted in twice higher odds of high antigenic dissimilarity between serum-virus pairs. This difference may have changed the epitope conformation in the virus, changing the affinity or avidity of antigen-antibody interaction. Site 39 in ORF5 forms part of the epitope region in PRRSV1 (20), and leucine-isoleucine substitutions in that site in highly pathogenic PRRSV2 (which is closely related to PRRSV1) in China were associated with changes in primary neutralizing epitope and delayed antibody response by the host (69). Similarly, tyrosine-histidine-arginine substitutions in site 79 of ORF3 (also significant in our data with a 0.8 to 2.2 odds ratio) have also been associated with changes in GP3-associated viral antigenicity for PRRSV2 (70). In summary, amino acid differences occurring in epitopes or regions close to epitopes influence pathogen antigenicity and host immune response, both of which are correlated with antigenic distance between isolates. Since detailed characterization of PRRSV1 has not been widely published, statistical associations between antigenic distance and particular amino acid sites identified in our study, combined with knowledge of the general structure of PRRSV, generate hypotheses about genetic determinants of cross-protection that can be further tested through experimental research.

Inferences from this analysis are influenced by some limitations in our study. Despite identifying important amino acid sites and differences between pairs associated with antigenic distance, our approach cannot ascertain causal relationships between the amino acid differences and the biological effects of those differences. Similarly, specific amino acid differences increased antigenic distance between serum-virus pairs. Although we know that different amino acids have specific biochemical properties (e.g., polar versus nonpolar, aromatic, etc.), we could not elucidate the biological impacts of those differences. While other methods such as mutagenesis are applicable to investigating cross-protection and amino acid interactions, our approach is inexpensive, and the predictive model can be used for *in silico* antigenic predictions for different strains, which is relevant for field-based disease management efforts. However, the role of specific sites and amino acid differences needs further exploration to better understand their biological significance.

All isolates used in this study belonged to one of four subtypes of PRRSV1 (subtype 1), and 63% of the isolates were from Spain. As a result, it is yet unclear whether our models can be generalized to other subtypes whose genetic profiles may still differ. In addition, we were only able to interrogate the impact of amino acid changes observed in our data; thus, our predictive model does not account for all changes in potentially antigenic sites that could exist in natural PRRSV1 populations but were not in our data. However, as demonstrated in Fig. 2, the predictive machine learning models did perform well when predicting immunogenic cross-reactivity for other field isolates that were dissimilar from the data used for model building, although these were still subtype 1. Since we used secondary data, we were unable to obtain an equal number of observations for all genome segments that reduced the number of compared pairs upon inclusion of ORF2 in the analysis, nor were we able to include ORFs 6 and 7, which also play a role in PRRSV immune responses (28, 71–75). Including these ORFs would provide a broader picture of the immune response landscape and cross-reactivity estimation. Finally, different factors (e.g., environment and host genetics) may influence PRRSV antigenicity and host immune response, particularly when translating *in vitro* cross-reactivity insights to *in vivo* cross-protection in the field; our model could not account for these factors.

### Conclusion.

Overall, this study reaffirms the association between viral antigenicity and genetic differences. We demonstrate that using machine learning techniques and sequence data, *in silico* estimation of antigenic distance of PRRSV1 can be achieved, although further research with diverse data sets could lead to more robust and generalizable models. Inferences of antigenic distance and possibly cross-protection from genetic sequence data using these methods present valuable opportunities to (i) reduce the cost and need for live animal/*in vivo* experiments for cross-protection/cross-neutralization assays; (ii) identify of immunodominant sites in different strains of PRRSV1 for further research; (iii) develop a prediction tool for *in silico* estimation of antigenic distance for tailoring immunization protocols in farms to match field outbreaks (i.e., selection of vaccine or live virus inoculum); and (iv) advance understanding of PRRSV1 ecology, trends of emergence, and immune escape. Additionally, providing the option to classify viruses based on comparative antigenic traits could complement the currently available PRRSV1 classification methods based on the genetic distance for a more accurate characterization of isolates. By identifying specific amino acid sites of more antigenic importance, we provide a basis for further investigation and characterization of the PRRSV1 genome and biological implications of specific amino acid substitutions in those sites.

## MATERIALS AND METHODS

### Data preparation.

For this analysis, we used seroneutralization (SN) assay outputs published by Martinez-Lobo et al. (20). In that study, they aimed to categorize PRRSV1 isolates circulating in Europe into clusters on the basis of susceptibility to neutralization. Antisera against 27 field isolates were generated using 6-month-old pigs inoculated with 5 mL of cell-cultured viruses via intramuscular and intranasal routes with appropriately spaced booster shots. These hyperimmune sera were serially diluted and cross-reacted with homologous and heterologous viruses in SN assays. Homologous sera titers were adjusted to 1:128 using Dulbecco’s modified Eagle’s medium as diluents or concentrating the serum immunoglobulins (Ig) (20, 76). Concentrated Ig from individual animals was used for the SN assays. Neutralization titers from the cross-neutralization of the 27 isolates and antisera were used for this analysis. The titers were expressed as log_2_ of the reciprocal of the serum dilution that completely inhibited viral replication in 50% of the wells. This values were recorded for each sera-virus pair in the SN panel (20). From the SN panel, we calculated antigenic distance by subtracting the log_2_ of heterologous titer from the log_2_ of homologous titers, i.e., *D_ij_ =* log _2_(*H_jj_*) – log _2_(*H_ij_*), with a one unit difference representing a 2-fold loss in neutralization ability between the homologous and heterologous pair (46, 64).

Subsequently, we downloaded the corresponding amino acid sequences published by Martinez-Lobo et al. (20) in GenBank (JF730883 to JF730921 for ORF3; JF730922 to JF730960 for ORF4 and JF730961 to JF730999 for ORF5). ORF2 sequences that were not part of the GenBank sequences have been submitted and assigned accession numbers (OP643789 to OP643820). We aligned the 27 amino acid sequences using Muscle in Aliview 1.26 (77) for ORFs 2 to 5 separately. These ORFs code for glycoproteins 2 to 5, respectively. Using MEGA X (78), we calculated pairwise amino acid p-distance and Poisson corrected amino acid distance between all pairs (78, 79) and coded differences at specific amino acid sites using R packages, *stringr* (80), *bioseq* (81), *ape* (82), and *tidysq* (83). Since the ectodomain of the different genes play a more critical role in antigen-antibody interactions and induction of immune response (84, 85), we based amino acid distances and site-wise amino acid differences on the ectodomains of each ORF: ORF2 (41:187), ORF3 (27:161), ORF4 (23:139), and ORF5 (32:64,100:109) (21, 22, 84). For each polymorphic amino acid site, sera-virus pairs were tabulated as 0 if they shared the same amino acid and 1 if they had a different amino acid at that site. Antigenic distance, amino acid distance, and amino acid site-wise differences were concatenated into one data frame. Four different ORF-specific data sets were generated for each ORF (2 to 5). Based on in initial descriptive analyses (see results), data sets were also prepared for three combinations of ORFs (2 to 5, 3 to 5, and 2 and 5). ORF2 data were only available for 19 of the 27 viruses (70%), thus models involving ORF2 had fewer pairs (324) for comparison than when modeling only data from ORF 3 to 5 (624). Site-wise amino acid differences between viral pairs were considered to be of equal importance and were thus unweighted (e.g., A→G ≡ G→A) (46). Similar studies in influenza suggest that weighting certain amino acid mutations may introduce noise to the analysis (86, 87). Correlation analysis between amino acid distance and antigenic distance was done and is summarized in tables and figures. For the purposes of visualization and ease of interpretation, frequency tables, and scatterplots were generated using the raw p-distance (78) rather than the Poisson-corrected distance (78, 79) (used in the models), although these values had a Pearson’s correlation of 0.99.

### Training and testing machine learning models.

Using modified mixed-effects supervised machine learning models (88), we accounted for serum-level random effects (i.e., sera appear in multiple sera-virus pairs, and some serum may be broadly neutralizing and thus have lower apparent antigenic distances across all sera-virus pairs [13]) by adjusting the antigenic distance value by subtracting the effect of specific sera from the antigenic distance. Following Wang et al. (88), we fit a general linear regression to the antigenic distance data using sera as a random effect; no fixed effects were included. The random-effect coefficients estimated for each serum were subtracted from the raw antigenic distance values to adjust for differences between serums. This was done to account for broadly or poorly neutralizing sera that may confound the predicted importance of certain amino acid differences due to repeated measurements.

Subsequently, we set up a gradient-boosting classification model (XGBoost [89]). While similar ML algorithms such as random forest were applicable to our data, when tested on part of the data (ORF5), XGboost performed better (area under the curve [AUC] = 0.74, Se = 0.7, Sp = 0.8) than random forest (AUC = 0.64, Se = 0.63, Sp = 0.66); therefore, we used XGboost to analyze the entire data set. The outcome of this model was adjusted antigenic distance, categorized into low and high groups using a median split. Predictor variables included Poisson corrected amino acid distance (78, 79) and site-wise amino acid differences. Amino acid distances were included as continuous variables while site-wise amino acid differences were fitted as categorical predictors in the model. Because we are repurposing data from an older study (20), the number of paired sera-virus observations for ORF2 (*n* = 314) was less than those available for ORFs 3 to 5 (*n* = 612) after excluding 10 and 12 pairs with negative antigenic distances, respectively. Negative antigenic distances occurred in some cases, indicating sera-virus pairs where the heterologous sera neutralized a virus better than the homologous sera. This could occur if the heterologous sera were broadly neutralizing, if the homologous serum had low avidity, or potentially lab error, although the last possibility is unlikely because all assays with unexpected results were repeated in a separate assay to confirm the data. These were excluded from further analysis. Compared to ORFs 3 and 4, slightly higher correlations between ectodomain amino acid distance and antigenic distance were observed for ORFs 2 and 5. Different combinations of the specific ORFs were used to build separate models and their performance was compared. The model combinations were ORFs 2 to 5, 3 to 5, and 2 and 5.

We used 200 trees and 10-fold cross-validation as part of the model tuning to train and make predictions with the model. We based model performance on accuracy (overall percentage of observations correctly classified as high/low), sensitivity (percentage of high observations correctly classified), specificity (percentage of low observations correctly classified), and predictive values (proportion [%] of times the classification [high or low] is the true antigenic distance class). Apart from model predictions, XGBoost models generate several other outputs. In this study, we used gain (i.e., the relative contribution to model accuracy of a predictor on the branches that it occurs) to rank the importance of model predictors (89). In our data, we considered the relative importance of different amino acid sites based on their role in improving model accuracy (46).

To test the performance of the machine learning algorithm’s predictions on a completely external set of sequences, we used data from Vanhee et al. (49). In this study, they evaluated three serum-virus pairs generated from the Lelystad vaccine prototype and two wild strain viruses (GenBank accession numbers GU737264 and GU737266) isolated form Belgian farms. The viruses were propagated in 19 naive wean piglets divided into groups of 6, 6, and 7, from which serum for neutralization assays was collected at day 44 postinoculation. Only ORF4 sequence data were available from this study, thus we used our ORF4-specific model to predict cross-neutralization across these serum-virus pairs and compared the predicted results to the experimental results reported in the paper.

### Quantifying the impact of amino acid differences.

Using variable importance rankings from the best XGBoost model, we selected the amino acid sites whose gain was estimated at ≥0.01 (21 sites) and the effect of these differences on antigenic dissimilarity was further analyzed via multimodal regression. We modeled the categorical pairwise antigenic distance as the outcome and amino acid sites as factor covariates to estimate the contribution of specific site-wise amino acid differences. Unlike in the above machine learning model (where site-wise differences were represented as 0: no difference, and 1: different), the specific amino acid changes were explored here (i.e., F-F versus F-S versus A-V versus V-A). For the purpose of logistic regression, the same-same amino acid pair with the highest frequency was set as the reference, and model coefficients can be interpreted as a comparator to the reference. We used exhaustive-screening multimodal logistic regression inference (2 million iterations) in *glmulti* package in R to select the best models. The best models were considered the simplest models that were ΔAICc ≤2 than the model with the lowest AICc (second-order Akaike information criterion correcting for small sample size). One hundred top ranked models (from 4 million iterations) based on AICc and Akaike weights were stored for comparison (90). Due to computational constraints, we used a two-step multimodal inference approach. In the first step, interactions between model covariates were excluded and the best-fit models (ΔAICc ≤ 2 of the model with the lowest AICc) were identified (Fig. S2), ranked, and weighted (Table S1). Model covariates were ranked based on the sum weights of the best-fit models in which they appeared (90), and all covariates with an overall variable importance of ≥0.5 were retained in the second step of multimodal inference (Fig. S3). In the second step, all covariates identified in step 1, and all possible interactions between the selected amino acid sites were included. From this analysis, the best-fit models (ΔAICc ≤2) were again selected based on AICc rankings (Fig. S4; Table S2). To estimate the effect of differences in amino acid residues in specific sites and their interactions on pairwise antigenic differences, model coefficients were averaged across all four models and interpreted as odds ratios using a significant *P* value cutoff of <0.05 (Table S3). Final summary statistics are reported for model covariates with an estimated importance of ≥0.8, and a full summary across all averaged models is presented in Table S4.

### Data availability.

The nucleotide sequences of the PRRSVs sequenced in the present study have been deposited in GenBank (OP643789-OP643820).
