# Supplementary material for: Predicting Antigenic Distance from Genetic Data for PRRSV-Type 1: Applications of Machine Learning
Source: Microbiol Spectr. 2022 Dec 13;11(1):e04085-22. doi: 10.1128/spectrum.04085-22 (PMC9927307; doi:10.1128/spectrum.04085-22)
Supplement: Supplemental file 1 — Fig. S1 to S4 and Tables S1 to S4. Download spectrum.04085-22-s0001.pdf, PDF file, 1.3 MB [file spectrum.04085-22-s0001.pdf]

Supplementary material

a)

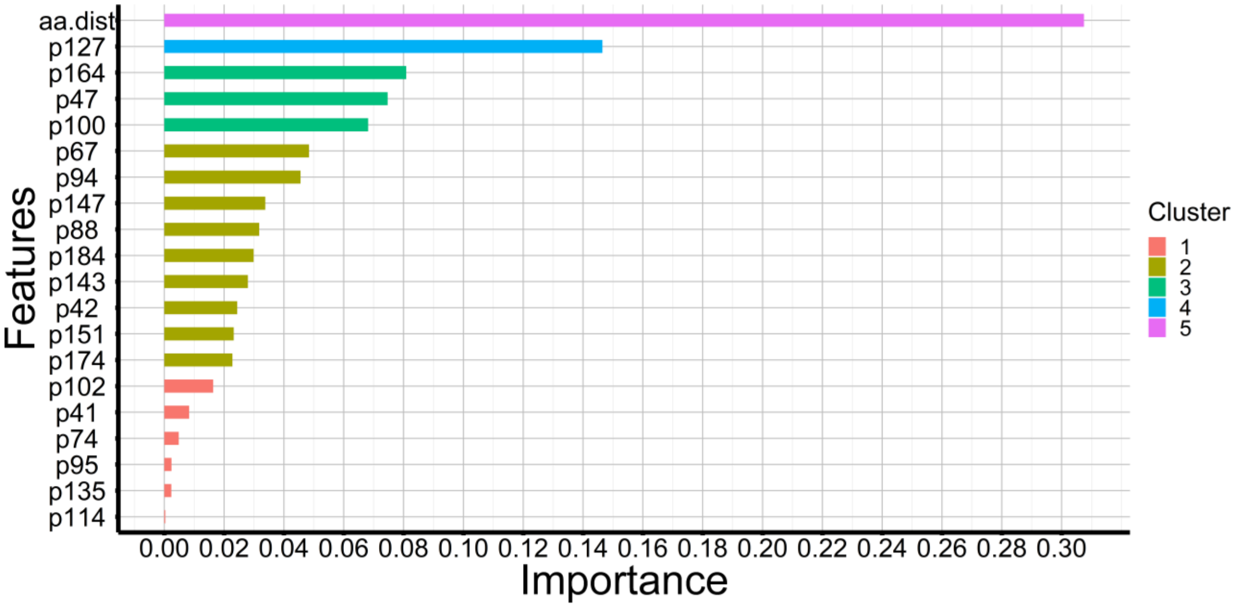

b)

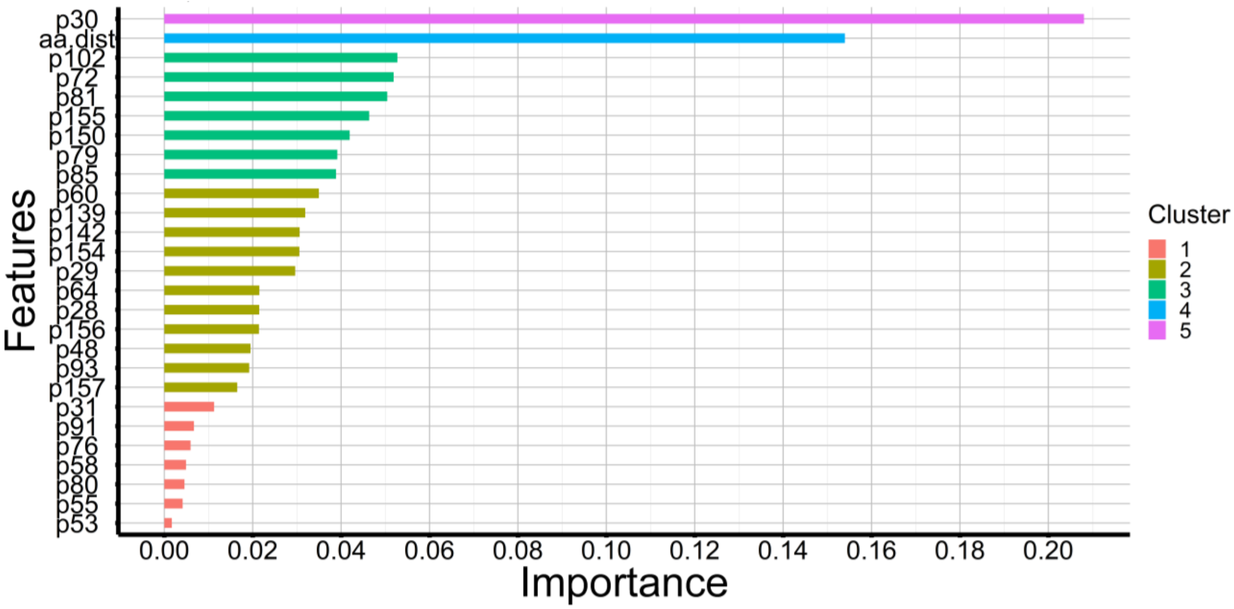

c)

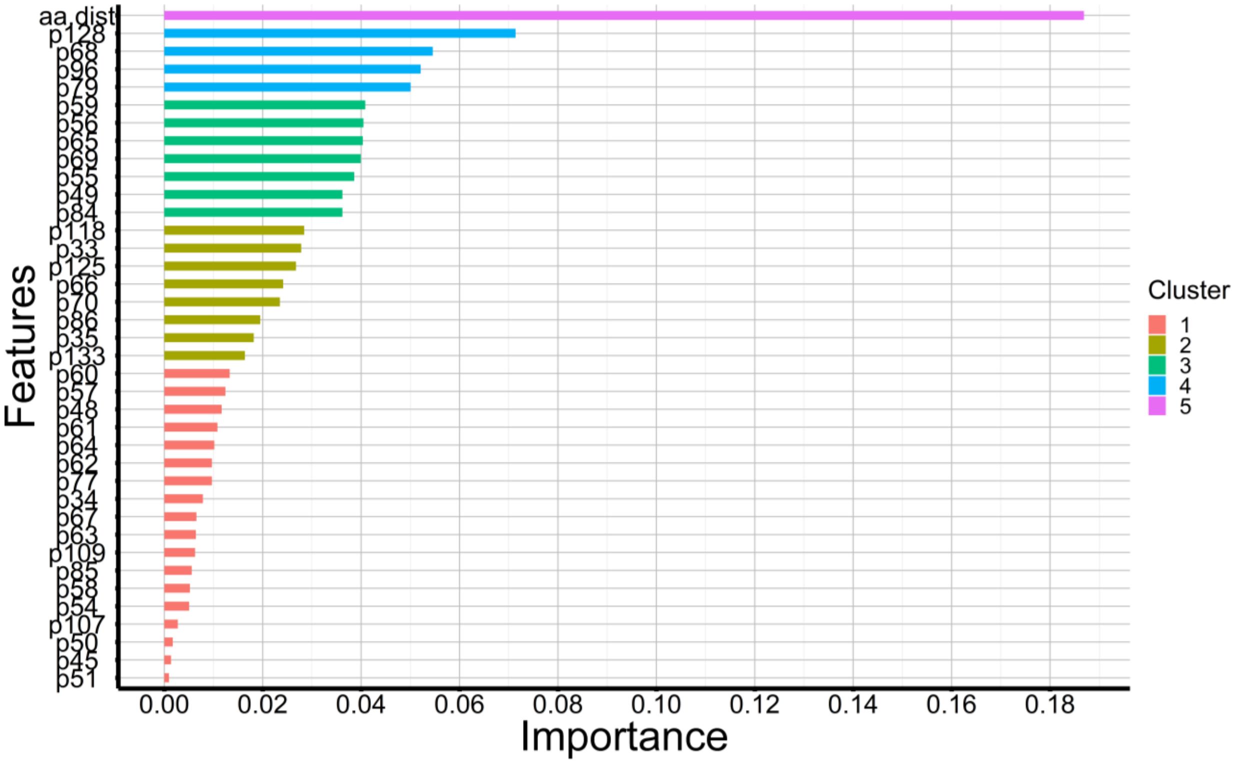
aa.dist
p128
p68
p96
p79
p59
p56
p65
p69
p55
p49
p84
p118
p33
p125
p66
p70
p86
p35
p133
p60
p57
p48
p61
p64
p62
p77
p34
p67
p63
p109
p85
p58
p54
p107
p50
p45
p51
Features
0.00 0.02 0.04 0.06 0.08 0.10 0.12 0.14 0.16 0.18
Importance
Cluster
1
2
3
4
5

d)

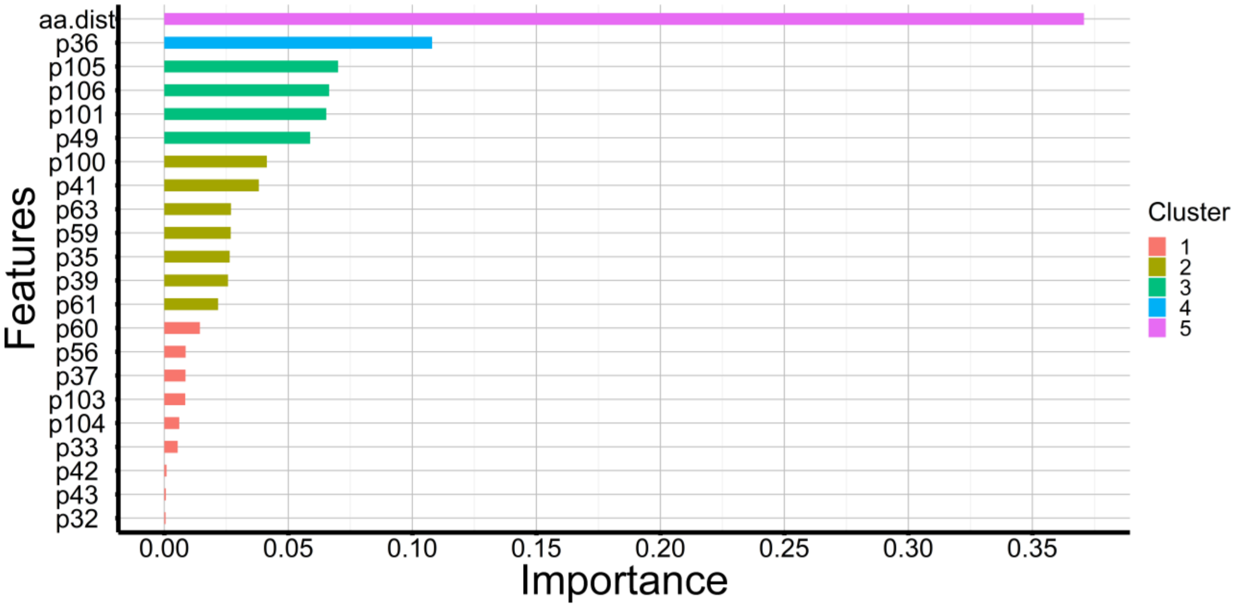
aa.dist
p36
p105
p106
p101
p49
p100
p41
p63
p59
p35
p39
p61
p60
p56
p37
p103
p104
p33
p42
p43
p32
Features
0.00 0.05 0.10 0.15 0.20 0.25 0.30 0.35
Importance
Cluster
1
2
3
4
5

e)

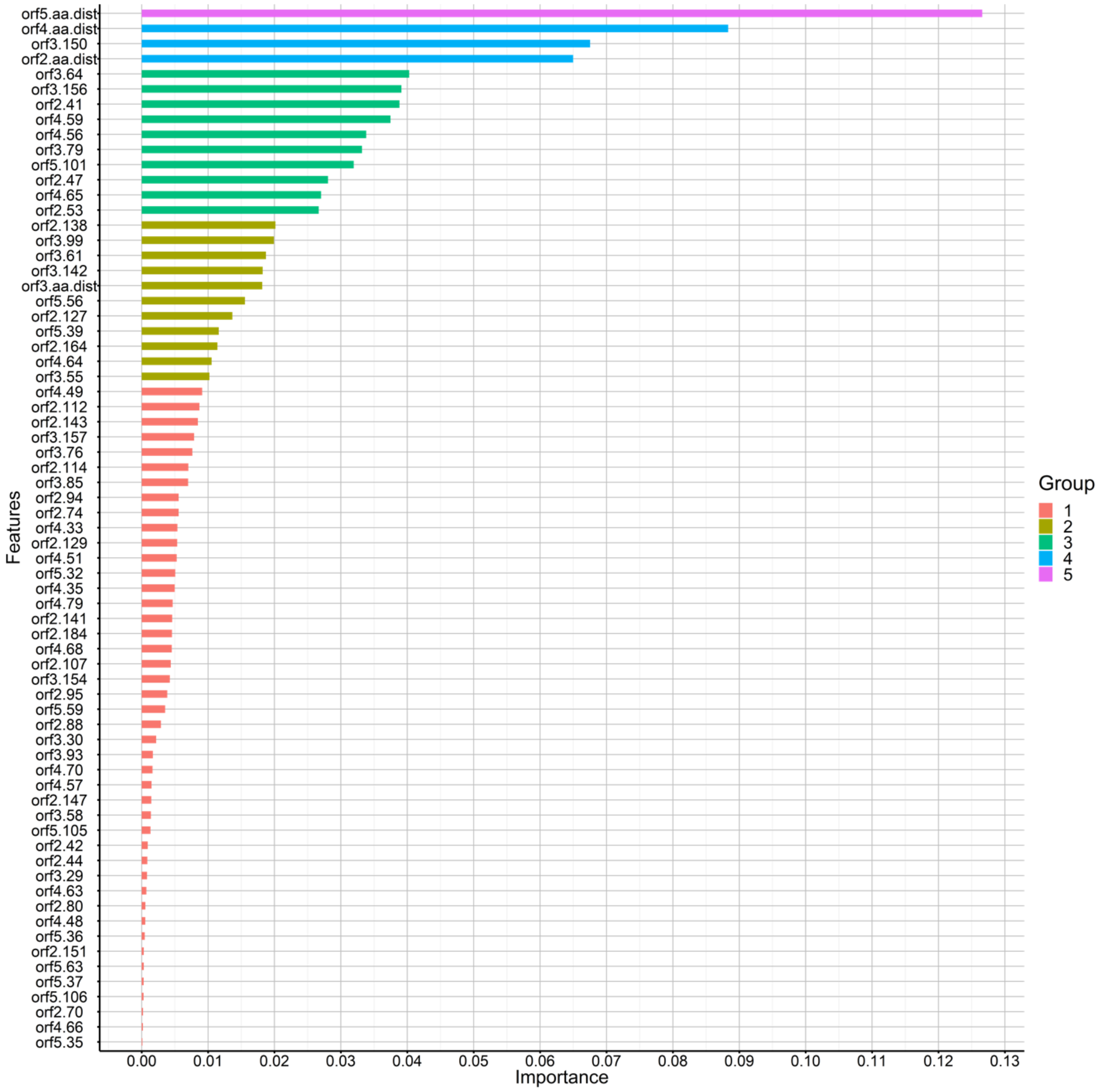

Figure 1 (a-e): XGBoost gain ranking of ORF specific model covariates: a) ORF2, b) ORF3, c) ORF4, d) ORF5, e) ORF 2-5. Feature labels (y-axis) are predictors in the models, with the prefix indicating the ORF and the suffix indicating either the amino acid in the ectodomain (aa.dist) or the specific amino acid site. Predictors are ranked based on overall importance considering gain (relative contribution of a variable to model accuracy).

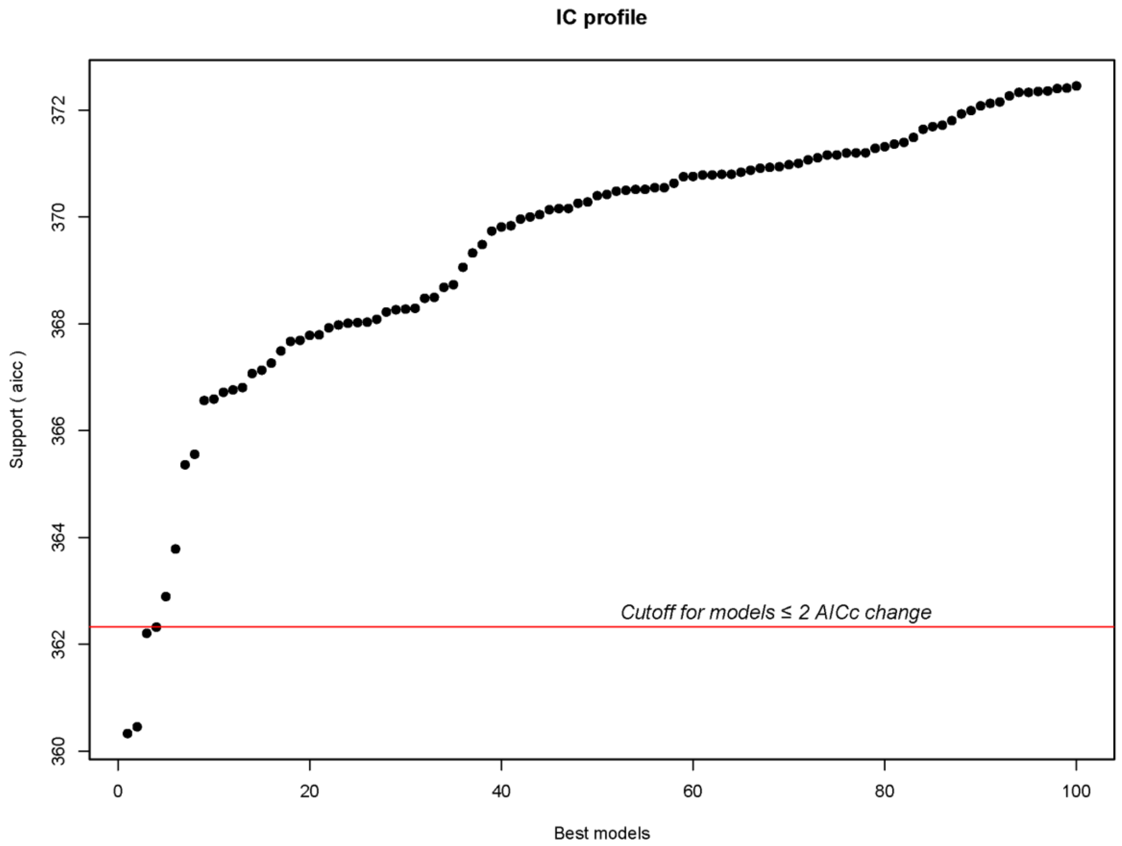

Figure 2: Information profile ranking of best models for analysis based on second order Akaike information criterion correcting for small sample size. Four models were selected for subsequent analysis.

	Model	AICc	Weights
1	adj.ag.dist2 ~ 1 + orf2.47 + orf2.127 + orf3.55 + orf3.79 + orf3.99 + orf3.142 + orf5.39	360.3	0.230
2	adj.ag.dist2 ~ 1 + orf2.47 + orf2.127 + orf2.164 + orf3.55 + orf3.79 + orf3.99 + orf3.142 + orf5.39	360.5	0.216
3	adj.ag.dist2 ~ 1 + orf2.47 + orf2.127 + orf3.55 + orf3.79 + orf3.99 + orf3.142 + orf5.39 + orf5.101	362.2	0.090
4	adj.ag.dist2 ~ 1 + orf2.47 + orf2.127 + orf2.164 + orf3.55 + orf3.79 + orf3.99 + orf3.142 + orf5.39 + orf5.101	362.3	0.085

Table 1: Summary of the five best models out of 100 models examined from initial multimodal logistic regression inference. Models are ranked based on the AICc and calculated Akaike weights. Models with higher weights are more suitable to explain the data.

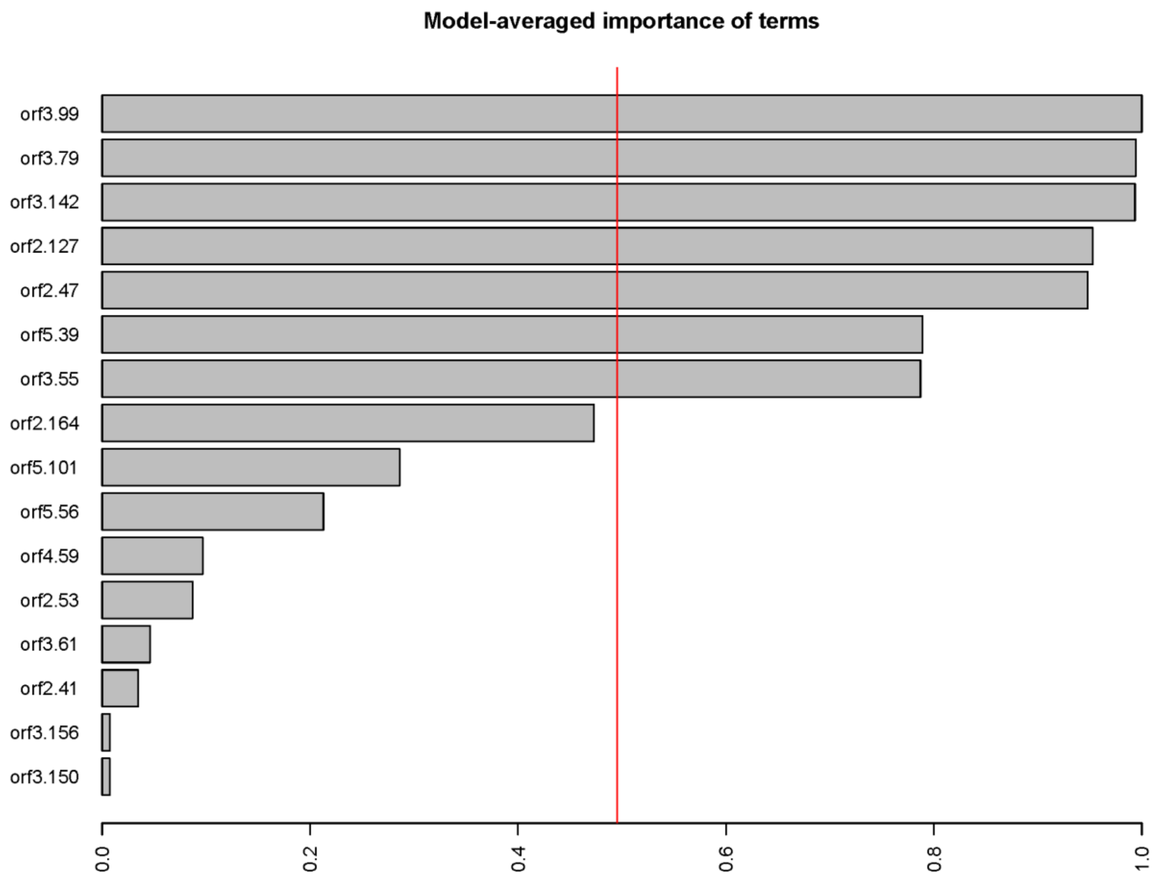

Figure 3: Estimated variable importance based on sum model weights in which the variables appear, also interpreted as the overall support of the model predictor across the candidate set of models. Predictors with overall importance of 0.5 were included in second step models

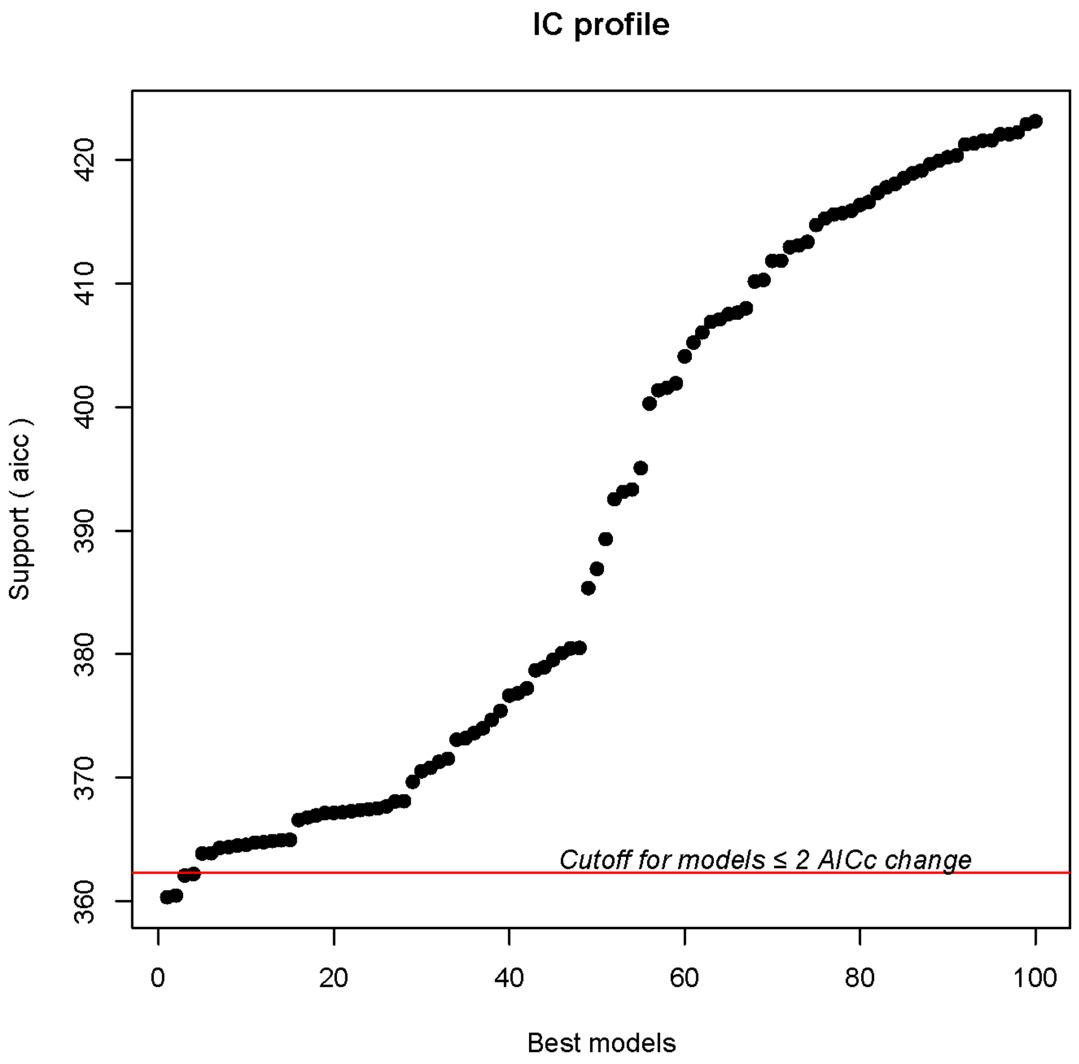

Figure 4: Information profile ranking of best models for analysis based on second order Akaike information criterion correcting for small sample size. Four models were selected for subsequent analysis.

	Model	AICc	Weights
1	adj.ag.dist2 ~ 1 + relevel(orf2.47, ref = "F|F") + relevel(orf2.127, ref = "V|V") + relevel(orf3.55, ref = "M|M") + relevel(orf3.79, ref = "H|H") + relevel(orf3.99, ref = "D|D") + relevel(orf3.142, ref = "F|F") + relevel(orf5.39, ref = "S|S")	360.3	0.219
2	adj.ag.dist2 ~ 1 + relevel(orf2.47, ref = "F|F") + relevel(orf2.127, ref = "V|V") + relevel(orf2.164, ref = "V|V") + relevel(orf3.55, ref = "M|M") + relevel(orf3.79, ref = "H|H") + relevel(orf3.99, ref = "D|D") + relevel(orf3.142, ref = "F|F") + relevel(orf5.39, ref = "S|S")	360.5	0.206
3	adj.ag.dist2 ~ 1 + relevel(orf2.47, ref = "F|F") + relevel(orf2.127, ref = "V|V") + relevel(orf3.55, ref = "M|M") + relevel(orf3.79, ref = "H|H") + relevel(orf3.99, ref = "D|D") + relevel(orf3.142, ref = "F|F") + relevel(orf5.39, ref = "S|S") + relevel(orf3.99, ref = "D|D"):relevel(orf3.55, ref = "M|M")	362.1	0.091
4	adj.ag.dist2 ~ 1 + relevel(orf2.47, ref = "F|F") + relevel(orf2.127, ref = "V|V") + relevel(orf2.164, ref = "V|V") + relevel(orf3.55, ref = "M|M") + relevel(orf3.79, ref = "H|H") + relevel(orf3.99, ref = "D|D") + relevel(orf3.142, ref = "F|F") + relevel(orf5.39, ref = "S|S") + relevel(orf3.99, ref = "D|D"):relevel(orf3.55, ref = "M|M")	362.2	0.086

Table 2: List of the four best models out of 100 models examined from final multimodal logistic regression inference. Models are ranked based on the AICc and calculated Akaike weights. Models with higher weights are more suitable to explain the data.

	Odds Ratio	Lower 95% CI	Upper 95% CI	Pr(>|z|)	Importance
(Intercept)	4.44	3.90	5.06	0.000	1
relevel(orf2.47, ref = "F|F")F|S	0.88	0.74	1.04	0.143	1
relevel(orf2.47, ref = "F|F")S|F	0.67	0.56	0.80	0.000	1
relevel(orf2.47, ref = "F|F")S|S	0.71	0.40	1.25	0.235	1
relevel(orf2.127, ref = "V|V")A|V	2.01	1.50	2.68	0.000	1
relevel(orf2.127, ref = "V|V")V|A	1.21	0.93	1.58	0.146	1
relevel(orf3.55, ref = "M|M")E|K	0.79	0.52	1.20	0.271	1
relevel(orf3.55, ref = "M|M")E|M	0.86	0.64	1.14	0.289	1
relevel(orf3.55, ref = "M|M")E|T	1.35	0.60	3.04	0.463	1

relevel(orf3.55, ref = "M|M")K|E	1.66	1.04	2.66	0.033	1
relevel(orf3.55, ref = "M|M")K|K	0.97	0.72	1.30	0.817	1
relevel(orf3.55, ref = "M|M")K|M	0.94	0.79	1.11	0.450	1
relevel(orf3.55, ref = "M|M")K|T	0.70	0.42	1.15	0.154	1
relevel(orf3.55, ref = "M|M")M|E	1.25	0.93	1.69	0.144	1
relevel(orf3.55, ref = "M|M")M|K	1.04	0.88	1.23	0.647	1
relevel(orf3.55, ref = "M|M")M|T	1.02	0.73	1.43	0.900	1
relevel(orf3.55, ref = "M|M")T|E	0.95	0.42	2.13	0.904	1
relevel(orf3.55, ref = "M|M")T|K	0.35	0.22	0.55	0.000	1
relevel(orf3.55, ref = "M|M")T|M	0.44	0.32	0.60	0.000	1
relevel(orf3.79, ref = "H|H")H|R	1.31	1.01	1.71	0.043	1
relevel(orf3.79, ref = "H|H")H|Y	0.94	0.79	1.12	0.504	1
relevel(orf3.79, ref = "H|H")R|H	0.95	0.71	1.26	0.717	1
relevel(orf3.79, ref = "H|H")R|Y	2.16	1.30	3.60	0.003	1
relevel(orf3.79, ref = "H|H")Y|H	0.79	0.66	0.95	0.012	1
relevel(orf3.79, ref = "H|H")Y|R	1.03	0.57	1.85	0.926	1
relevel(orf3.79, ref = "H|H")Y|Y	0.70	0.42	1.16	0.164	1
relevel(orf3.99, ref = "D|D")D|E	1.20	0.98	1.48	0.077	1
relevel(orf3.99, ref = "D|D")E|D	0.79	0.62	1.00	0.051	1
relevel(orf3.99, ref = "D|D")E|E	0.60	0.44	0.81	0.001	1
relevel(orf3.142, ref = "F|F")C|F	0.59	0.47	0.74	0.000	1
relevel(orf3.142, ref = "F|F")C|V	1.70	0.75	3.82	0.201	1
relevel(orf3.142, ref = "F|F")F|C	1.01	0.80	1.26	0.951	1
relevel(orf3.142, ref = "F|F")F|V	1.03	0.77	1.37	0.862	1
relevel(orf5.39, ref = "S|S")L|L	2.52	1.31	4.85	0.006	1
relevel(orf5.39, ref = "S|S")L|S	2.23	1.67	2.97	0.000	1
relevel(orf5.39, ref = "S|S")S|L	1.03	0.79	1.35	0.813	1
relevel(orf2.164, ref = "V|V")A|V	0.89	0.65	1.21	0.446	0.4313
relevel(orf2.164, ref = "V|V")V|A	1.02	0.89	1.17	0.798	0.4313
relevel(orf3.55, ref = "M|M")E|M:relevel(orf3.99, ref = "D|D")D|E	1.12	0.77	1.62	0.559	0.1963
relevel(orf3.55, ref = "M|M")K|M:relevel(orf3.99, ref = "D|D")D|E	1.01	0.93	1.09	0.804	0.1963

relevel(orf3.55, ref = "M|M")M|T:relevel(orf3.99, ref = "D|D")D|E	0.97	0.83	1.14	0.709	0.1963
relevel(orf3.55, ref = "M|M")M|E:relevel(orf3.99, ref = "D|D")E|D	0.90	0.62	1.29	0.562	0.1963
relevel(orf3.55, ref = "M|M")M|K:relevel(orf3.99, ref = "D|D")E|D	0.98	0.89	1.08	0.683	0.1963
relevel(orf3.55, ref = "M|M")T|M:relevel(orf3.99, ref = "D|D")E|D	1.02	0.89	1.17	0.762	0.1963
relevel(orf3.142, ref = "F|F")C|F:relevel(orf3.99, ref = "D|D")D|E	0.99	0.95	1.04	0.744	0.063
relevel(orf3.142, ref = "F|F")F|C:relevel(orf3.99, ref = "D|D")E|D	1.02	0.94	1.11	0.630	0.063
relevel(orf3.142, ref = "F|F")F|V:relevel(orf3.99, ref = "D|D")E|D	1.01	0.95	1.07	0.679	0.063
relevel(orf2.127, ref = "V|V")A|V:relevel(orf2.47, ref = "F|F")F|S	1.02	0.94	1.10	0.683	0.0614
relevel(orf2.127, ref = "V|V")V|A:relevel(orf2.47, ref = "F|F")S|F	1.01	0.95	1.08	0.734	0.0614
relevel(orf2.127, ref = "V|V")V|A:relevel(orf3.99, ref = "D|D")D|E	1.01	0.96	1.06	0.699	0.0502
relevel(orf2.127, ref = "V|V")A|V:relevel(orf3.99, ref = "D|D")E|D	1.00	0.97	1.03	0.863	0.0502
relevel(orf3.99, ref = "D|D")E|D:relevel(orf5.39, ref = "S|S")L|S	1.00	0.97	1.02	0.787	0.0413
relevel(orf3.99, ref = "D|D")D|E:relevel(orf5.39, ref = "S|S")S|L	0.99	0.94	1.04	0.653	0.0413
relevel(orf2.127, ref = "V|V")V|A:relevel(orf5.39, ref = "S|S")L|S	1.00	0.97	1.03	0.849	0.0412
relevel(orf2.127, ref = "V|V")A|V:relevel(orf5.39, ref = "S|S")S|L	1.02	0.95	1.09	0.656	0.0412
relevel(orf2.47, ref = "F|F")S|F:relevel(orf3.99, ref = "D|D")D|E	1.00	0.98	1.01	0.712	0.0266
relevel(orf2.47, ref = "F|F")F|S:relevel(orf3.99, ref = "D|D")E|D	1.00	0.99	1.01	0.788	0.0266
relevel(orf2.164, ref = "V|V")V|A:relevel(orf5.39, ref = "S|S")L|S	1.00	0.98	1.03	0.779	0.0251
relevel(orf2.164, ref = "V|V")A|V:relevel(orf5.39, ref = "S|S")S|L	1.01	0.97	1.04	0.699	0.0251
relevel(orf2.47, ref = "F|F")F|S:relevel(orf5.39, ref = "S|S")L|S	1.00	0.99	1.01	0.932	0.0243
relevel(orf2.47, ref = "F|F")S|F:relevel(orf5.39, ref = "S|S")S|L	1.00	0.97	1.02	0.709	0.0243

Table 3: Summary of averaged 4 best models (AICc ≤ 2) out of the top 100 models examined from final multimodal logistic regression inference. Model covariates were ranked based on the sum weights of the models in which they appeared.

Genome section	Amino acid site	Amino acid residues	Site specific N.	Site specific percent %
orf2	41	P vs P	1	0.3
orf2	41	P vs Q	16	5.1
orf2	41	Q vs P	34	10.8
orf2	41	Q vs Q	263	83.8
orf2	47	F vs F	252	80.3
orf2	47	F vs S	32	10.2
orf2	47	S vs F	28	8.9
orf2	47	S vs S	2	0.6
orf2	53	A vs A	247	78.7
orf2	53	A vs V	31	9.9
orf2	53	V vs A	34	10.8
orf2	53	V vs V	2	0.6
orf2	127	A vs V	17	5.4
orf2	127	V vs A	17	5.4
orf2	127	V vs V	280	89.2
orf2	138	E vs E	2	0.6
orf2	138	E vs G	22	7
orf2	138	E vs K	2	0.6
orf2	138	E vs Q	4	1.3
orf2	138	E vs R	6	1.9
orf2	138	G vs E	21	6.7
orf2	138	G vs G	107	34.1
orf2	138	G vs K	11	3.5
orf2	138	G vs Q	22	7
orf2	138	G vs R	33	10.5
orf2	138	K vs E	2	0.6
orf2	138	K vs G	11	3.5
orf2	138	K vs Q	2	0.6
orf2	138	K vs R	3	1
orf2	138	Q vs E	2	0.6
orf2	138	Q vs G	11	3.5
orf2	138	Q vs K	1	0.3
orf2	138	Q vs Q	1	0.3
orf2	138	Q vs R	3	1
orf2	138	R vs E	5	1.6
orf2	138	R vs G	30	9.6
orf2	138	R vs K	2	0.6
orf2	138	R vs Q	6	1.9
orf2	138	R vs R	5	1.6
orf2	164	A vs V	18	5.7
orf2	164	V vs A	17	5.4

orf2	164	V vs V	279	88.9
orf3	55	E vs K	4	1.3
orf3	55	E vs M	13	4.1
orf3	55	E vs T	1	0.3
orf3	55	K vs E	3	1
orf3	55	K vs K	9	2.9
orf3	55	K vs M	39	12.4
orf3	55	K vs T	3	1
orf3	55	M vs E	12	3.8
orf3	55	M vs K	50	15.9
orf3	55	M vs M	150	47.8
orf3	55	M vs T	12	3.8
orf3	55	T vs E	1	0.3
orf3	55	T vs K	4	1.3
orf3	55	T vs M	13	4.1
orf3	61	H vs H	1	0.3
orf3	61	H vs Q	17	5.4
orf3	61	Q vs H	33	10.5
orf3	61	Q vs Q	263	83.8
orf3	64	E vs E	3	1
orf3	64	E vs H	4	1.3
orf3	64	E vs N	2	0.6
orf3	64	E vs Q	6	1.9
orf3	64	E vs R	9	2.9
orf3	64	E vs S	8	2.5
orf3	64	E vs Y	1	0.3
orf3	64	H vs E	6	1.9
orf3	64	H vs H	2	0.6
orf3	64	H vs N	2	0.6
orf3	64	H vs Q	6	1.9
orf3	64	H vs R	10	3.2
orf3	64	H vs S	8	2.5
orf3	64	H vs Y	2	0.6
orf3	64	N vs E	3	1
orf3	64	N vs H	2	0.6
orf3	64	N vs Q	3	1
orf3	64	N vs R	5	1.6
orf3	64	N vs S	3	1
orf3	64	N vs Y	1	0.3
orf3	64	Q vs E	9	2.9
orf3	64	Q vs H	6	1.9
orf3	64	Q vs N	3	1
orf3	64	Q vs Q	6	1.9

orf3	64	Q vs R	15	4.8
orf3	64	Q vs S	11	3.5
orf3	64	Q vs Y	3	1
orf3	64	R vs E	14	4.5
orf3	64	R vs H	9	2.9
orf3	64	R vs N	5	1.6
orf3	64	R vs Q	15	4.8
orf3	64	R vs R	17	5.4
orf3	64	R vs S	20	6.4
orf3	64	R vs Y	5	1.6
orf3	64	S vs E	12	3.8
orf3	64	S vs H	8	2.5
orf3	64	S vs N	4	1.3
orf3	64	S vs Q	12	3.8
orf3	64	S vs R	20	6.4
orf3	64	S vs S	12	3.8
orf3	64	S vs Y	4	1.3
orf3	64	Y vs E	3	1
orf3	64	Y vs H	2	0.6
orf3	64	Y vs N	1	0.3
orf3	64	Y vs Q	3	1
orf3	64	Y vs R	5	1.6
orf3	64	Y vs S	4	1.3
orf3	79	H vs H	207	65.9
orf3	79	H vs R	14	4.5
orf3	79	H vs Y	44	14
orf3	79	R vs H	15	4.8
orf3	79	R vs Y	3	1
orf3	79	Y vs H	26	8.3
orf3	79	Y vs R	2	0.6
orf3	79	Y vs Y	3	1
orf3	99	D vs D	136	43.3
orf3	99	D vs E	71	22.6
orf3	99	E vs D	78	24.8
orf3	99	E vs E	29	9.2
orf3	142	C vs F	14	4.5
orf3	142	C vs V	1	0.3
orf3	142	F vs C	16	5.1
orf3	142	F vs F	266	84.7
orf3	142	F vs V	17	5.4
orf3	15	H vs Q	12	3.8
orf3	15	H vs R	1	0.3
orf3	15	Q vs H	15	4.8

orf3	15	Q vs Q	223	71
orf3	15	Q vs R	30	9.6
orf3	15	R vs H	2	0.6
orf3	15	R vs Q	29	9.2
orf3	15	R vs R	2	0.6
orf3	156	A vs A	2	0.6
orf3	156	A vs N	2	0.6
orf3	156	A vs T	32	10.2
orf3	156	N vs A	2	0.6
orf3	156	N vs T	16	5.1
orf3	156	T vs A	29	9.2
orf3	156	T vs N	14	4.5
orf3	156	T vs T	217	69.1
orf4	56	H vs H	191	60.8
orf4	56	H vs P	14	4.5
orf4	56	H vs Q	14	4.5
orf4	56	H vs R	13	4.1
orf4	56	H vs Y	14	4.5
orf4	56	P vs H	14	4.5
orf4	56	P vs Q	1	0.3
orf4	56	P vs R	1	0.3
orf4	56	P vs Y	1	0.3
orf4	56	Q vs H	15	4.8
orf4	56	Q vs P	1	0.3
orf4	56	Q vs R	1	0.3
orf4	56	Q vs Y	1	0.3
orf4	56	R vs H	13	4.1
orf4	56	R vs P	1	0.3
orf4	56	R vs Q	0	0
orf4	56	R vs Y	1	0.3
orf4	56	Y vs H	15	4.8
orf4	56	Y vs P	1	0.3
orf4	56	Y vs Q	1	0.3
orf4	56	Y vs R	1	0.3
orf4	59	P vs P	2	0.6
orf4	59	P vs S	33	10.5
orf4	59	S vs P	32	10.2
orf4	59	S vs S	247	78.7
orf4	64	A vs E	1	0.3
orf4	64	A vs G	1	0.3
orf4	64	A vs I	1	0.3
orf4	64	A vs K	2	0.6
orf4	64	A vs N	7	2.2

orf4	64	A vs S	2	0.6
orf4	64	A vs T	3	1
orf4	64	A vs V	1	0.3
orf4	64	E vs A	1	0.3
orf4	64	E vs G	1	0.3
orf4	64	E vs I	1	0.3
orf4	64	E vs K	2	0.6
orf4	64	E vs N	7	2.2
orf4	64	E vs S	2	0.6
orf4	64	E vs T	3	1
orf4	64	E vs V	1	0.3
orf4	64	G vs A	1	0.3
orf4	64	G vs E	1	0.3
orf4	64	G vs I	1	0.3
orf4	64	G vs K	2	0.6
orf4	64	G vs N	7	2.2
orf4	64	G vs S	2	0.6
orf4	64	G vs T	3	1
orf4	64	G vs V	1	0.3
orf4	64	I vs A	1	0.3
orf4	64	I vs E	1	0.3
orf4	64	I vs G	1	0.3
orf4	64	I vs K	2	0.6
orf4	64	I vs N	7	2.2
orf4	64	I vs S	2	0.6
orf4	64	I vs T	3	1
orf4	64	I vs V	1	0.3
orf4	64	K vs A	2	0.6
orf4	64	K vs E	2	0.6
orf4	64	K vs G	1	0.3
orf4	64	K vs I	2	0.6
orf4	64	K vs K	1	0.3
orf4	64	K vs N	13	4.1
orf4	64	K vs S	3	1
orf4	64	K vs T	5	1.6
orf4	64	K vs V	2	0.6
orf4	64	N vs A	7	2.2
orf4	64	N vs E	7	2.2
orf4	64	N vs G	7	2.2
orf4	64	N vs I	7	2.2
orf4	64	N vs K	14	4.5
orf4	64	N vs N	42	13.4
orf4	64	N vs S	14	4.5

orf4	64	N vs T	20	6.4
orf4	64	N vs V	7	2.2
orf4	64	S vs A	1	0.3
orf4	64	S vs E	0	0
orf4	64	S vs G	1	0.3
orf4	64	S vs I	1	0.3
orf4	64	S vs K	2	0.6
orf4	64	S vs N	5	1.6
orf4	64	S vs S	1	0.3
orf4	64	S vs T	3	1
orf4	64	S vs V	1	0.3
orf4	64	T vs A	3	1
orf4	64	T vs E	3	1
orf4	64	T vs G	3	1
orf4	64	T vs I	3	1
orf4	64	T vs K	6	1.9
orf4	64	T vs N	21	6.7
orf4	64	T vs S	6	1.9
orf4	64	T vs T	6	1.9
orf4	64	T vs V	3	1
orf4	64	V vs A	1	0.3
orf4	64	V vs E	1	0.3
orf4	64	V vs G	1	0.3
orf4	64	V vs I	1	0.3
orf4	64	V vs K	2	0.6
orf4	64	V vs N	6	1.9
orf4	64	V vs S	2	0.6
orf4	64	V vs T	3	1
orf4	65	- vs -	6	1.9
orf4	65	- vs A	3	1
orf4	65	- vs G	6	1.9
orf4	65	- vs I	17	5.4
orf4	65	- vs S	6	1.9
orf4	65	- vs T	12	3.8
orf4	65	- vs V	3	1
orf4	65	A vs -	2	0.6
orf4	65	A vs G	2	0.6
orf4	65	A vs I	6	1.9
orf4	65	A vs S	1	0.3
orf4	65	A vs T	3	1
orf4	65	A vs V	1	0.3
orf4	65	G vs -	3	1
orf4	65	G vs A	1	0.3

orf4	65	G vs G	1	0.3
orf4	65	G vs I	6	1.9
orf4	65	G vs S	2	0.6
orf4	65	G vs T	4	1.3
orf4	65	G vs V	1	0.3
orf4	65	I vs -	18	5.7
orf4	65	I vs A	5	1.6
orf4	65	I vs G	11	3.5
orf4	65	I vs I	30	9.6
orf4	65	I vs S	12	3.8
orf4	65	I vs T	21	6.7
orf4	65	I vs V	6	1.9
orf4	65	S vs -	6	1.9
orf4	65	S vs A	2	0.6
orf4	65	S vs G	4	1.3
orf4	65	S vs I	12	3.8
orf4	65	S vs S	2	0.6
orf4	65	S vs T	8	2.5
orf4	65	S vs V	2	0.6
orf4	65	T vs -	12	3.8
orf4	65	T vs A	4	1.3
orf4	65	T vs G	8	2.5
orf4	65	T vs I	24	7.6
orf4	65	T vs S	8	2.5
orf4	65	T vs T	12	3.8
orf4	65	T vs V	4	1.3
orf4	65	V vs -	3	1
orf4	65	V vs A	1	0.3
orf4	65	V vs G	2	0.6
orf4	65	V vs I	5	1.6
orf4	65	V vs S	2	0.6
orf4	65	V vs T	4	1.3
orf5	39	L vs L	2	0.6
orf5	39	L vs S	34	10.8
orf5	39	S vs L	31	9.9
orf5	39	S vs S	247	78.7
orf5	56	A vs A	9	2.9
orf5	56	A vs D	15	4.8
orf5	56	A vs E	23	7.3
orf5	56	A vs H	3	1
orf5	56	A vs T	3	1
orf5	56	D vs A	19	6.1
orf5	56	D vs D	17	5.4

orf5	56	D vs E	37	11.8
orf5	56	D vs H	4	1.3
orf5	56	D vs T	5	1.6
orf5	56	E vs A	32	10.2
orf5	56	E vs D	40	12.7
orf5	56	E vs E	55	17.5
orf5	56	E vs H	8	2.5
orf5	56	E vs T	8	2.5
orf5	56	H vs A	4	1.3
orf5	56	H vs D	5	1.6
orf5	56	H vs E	8	2.5
orf5	56	H vs T	1	0.3
orf5	56	T vs A	4	1.3
orf5	56	T vs D	5	1.6
orf5	56	T vs E	8	2.5
orf5	56	T vs H	1	0.3
orf5	101	A vs A	13	4.1
orf5	101	A vs I	8	2.5
orf5	101	A vs T	46	14.6
orf5	101	I vs A	10	3.2
orf5	101	I vs I	2	0.6
orf5	101	I vs T	23	7.3
orf5	101	T vs A	59	18.8
orf5	101	T vs I	23	7.3
orf5	101	T vs T	130	41.4

Table 4: Frequency of amino acid pairs compared in multimodal inference in ORFs 2-5 ectodomains for the top 21 amino acid sites selected based on gradient boositng gain.
